# Bibliometric Analysis of Traditional Chinese Medicine Scientific Production between 1982 and 2016 Indexed in PubMed

**DOI:** 10.3390/medicines5020041

**Published:** 2018-05-03

**Authors:** Ricardo Consentino, Maria João Santos, Luís Carlos Matos, Jorge Pereira Machado

**Affiliations:** 1 ICBAS—Institute of Biomedical Sciences Abel Salazar, University of Porto, 4050-313 Porto, Portugal; rconsentino@yahoo.com (R.C.); mjrs.mtc@gmail.com (M.J.S.); 2 Faculdade de Engenharia da Universidade do Porto, Rua Dr. Roberto Frias, 4200-465 Porto, Portugal; lcmatos@fe.up.pt; 3 LABIOMEP—Porto Biomechanics Laboratory, University of Porto, 4200-450 Porto, Portugal

**Keywords:** acupuncture, traditional Chinese medicine, bibliometric analysis, PubMed

## Abstract

**Background:** Traditional Chinese medicine (TCM) may be understood as a system of sensations and findings designed to establish the functional vegetative state of the body. This state may be treated by several therapeutic methods such as acupuncture, Chinese pharmacotherapy, dietetics, *Tuina*, and *Qigong*. Nowadays, as a result of several evidence-based reported beneficial effects over specific pathological conditions, there is an increasing tendency to integrate some of these practices in Western medicine. The main goal of this study was to perform a bibliometric analysis of TCM scientific production between 1982 and 2016 indexed in PubMed, by analyzing several parameters including time and location distribution, publication quality, experimental design, and treatment methods. **Methods:** The methodology was based on the quantitative inventory of published scientific research indexed in PubMed medical subject headings (MeSH), sorted within the broad term “Traditional Chinese Medicine” and integrating the following criteria as limit filters: “Species: Humans”, “Article Type: Clinical Trial”. In addition, the articles’ triage was ruled by temporal limitations set between 1945 and 2016. **Results:** The overall analysis of data allowed observation of an average annual growth of approximately 33%, with a productive peak of 122 articles in 2007. The scientific production was distributed in 27 countries, led by China (76.1%), followed by the United States of America (3.0%) and South Korea (2.1%). A significant amount of references were published in Chinese journals: more than 50%; however, these journals had a low impact factor. The most cited treatments in the keywords section of the articles were phytotherapy (55%) and acupuncture (40%). **Conclusion:** The increasing demand for TCM seems to be due to factors such as lower side effects and greater efficacy in some patients not responding well to conventional therapy. As a result, a considerable amount of TCM science-based literature has been produced, supporting the rational integration of these practices in Western healthcare systems and research. Our results show that the quality of TCM research and inherent publications have been increasing over the last decades, with a higher incidence of studies published in well-ranked journals.

## 1. Introduction

Traditional Chinese medicine (TCM) may be understood as a system of sensations and findings designed to establish the functional vegetative state of the body [1,2]. It is driven by a unique theoretical paradigm with key concepts and theories such as the *Yin/Yang*, the “Five Elements”, the elemental substances *Qi*, *Xue*, and *Jin Ye*, as well as the approach to differential diagnosis of syndromes. Treatment methodology may include the combined or standalone use of acupuncture; moxibustion; Chinese pharmacotherapy; Chinese manual therapy, known as *Tuina*; and traditional biofeedback exercises such as *Qigong* and *Tai Chi* [3,4,5,6,7,8].

The worldwide increasing interest in traditional and complementary medicine and the proportional scientific production in this field encouraged the World Health Organization (WHO) to advocate the inclusion of some of these practices in the national healthcare systems, with the purpose of improving service quality and accessibility as well as cost reduction. It is also recommended to the governments of each country to establish mechanisms for licensing and accreditation of TCM [9,10]. In fact, some European countries, Portugal among them, have already produced and approved legislation that establishes the required conditions to become an official TCM practitioner with a government-approved professional license. This legislation defines the professional profile and inherent graduation syllabus [11,12]. The courage shown by approving and implementing this legal framework set Portugal in the vanguard of complementary and integrative medicine in Europe.

One way to monitor and analyze the trends of TCM advances and difficulties in the West is through scientometrics. This approach stands on the quantitative analysis of TCM scientific production through the use of bibliometric tools.

Currently, scientometrics is widely used to systematically evaluate scientific performance and to support strategic decision making such as the attribution of grants and allocation of technical resources [13,14,15]. Bibliometric analysis is commonly used to refresh the status quo in several areas of Western medicine; however in TCM, its use is still considerable less expressive. Within this scenario, studies revealing the status of TMC scientific production are essential to find out how and where TMC knowledge is distributed, as well as to design local policies to improve it.

Several bibliometric studies on TCM scientific production have been published in the past years [16,17,18,19]. Those studies point out to an increasing tendency to conduct TCM research over the last decades. Those studies also reveal that the scientific community is interested in TCM therapeutic methods such as phytotherapy and acupuncture. This increasing interest might be due, among other factors, to the reported effectiveness of these practices in the treatment of certain conditions, and the near absence of side effects. Nevertheless, the rational integration of TCM in Western healthcare systems and research requires the objectivation of TCM practices with a clear science-based approach incorporated from the theoretical foundations to the treatment procedures. In this process, the publication of scientific peer-reviewed articles in well-rated journals is essential to breakdown any doubt, resistance, or ambiguity.

The main goal of this study was to perform a bibliometric analysis of the world’s scientific production in traditional Chinese medicine between the years of 1982 and 2016, indexed in PubMed, and to analyze the chronological and geographical distribution based on the author’s institutional affiliation, and publication quality, study design, and treatment methods mentioned in the keywords section of the selected studies.

## 2. Materials and Methods

The TCM scientific production inventory was carried out by electronic survey in October 2016. The PubMed database was assessed and only the publications indexed in Medical Subject Headings (MeSH) were selected, sorted within the broad term “Traditional Chinese Medicine” and integrating the following criteria as limit filters: “Species: Humans” and “Article Type: Clinical Trial”. As well, the articles’ triage was also ruled by temporal limitations set between 1945 and 2016.

Data were analyzed according to the following indicators: chronological distribution; geographical distribution by country based on the author’s institutional affiliation; publication quality assessed through impact factor analysis on the ResearchGate platform; study design and treatment methods mentioned in the keywords section of the articles.

Reference Manager 12 Demo software and Microsoft Excel were used to manage data, and basic descriptive statistics was used.

## 3. Results

A total of 1072 scientific articles were found in this survey. There was no record of publications prior to 1982.

### 3.1. Chronological Evolution of TCM Scientific Production

**Figure 1:**
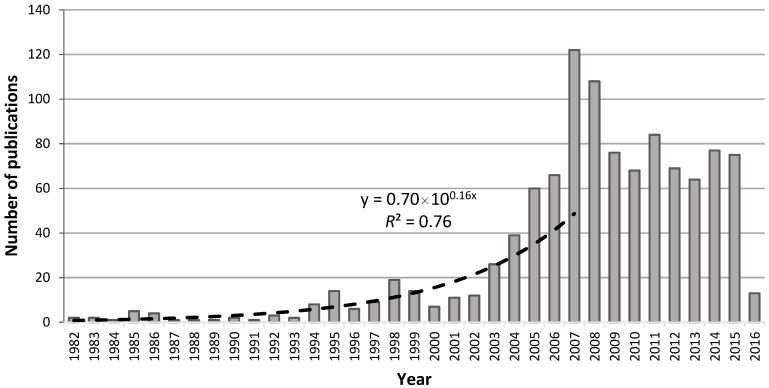
Evolution of published traditional Chinese medicine (TCM) research in the period 1982–2016 (dashed line represents the exponential tendency between 1982 (x = 1) and 2007 (x = 26)). *R*^2^ is the coefficient of determination, which gives the percentage variation in “*y*” explained by the variable “*x*”.

There was an exponential growth of scientific production between 1982 and 2007, with an average annual growth of approximately 33%, and a peak production of 122 articles in 2007 (see Figure 1).

The previous mentioned tendency was assessed by fitting an exponential function to experimental data, which allowed us to verify a high coefficient of determination (*R*^2^ = 0.76) and inherent coefficient of correlation (*R* = 0.87), indicating that the data evolution over time is well predicted by the chosen model. Additionally, a closer analysis of Figure 1 shows the possible existence of four distinct periods. Indeed, from 1982 to 1993, an unexpressive steady-state tendency is noticed, followed by a period, until 2000, of slight increase of scientific production. The year 2000 marked the beginning of a period of rapid growth, with a peak reached in 2007. From this year on, the tendency decreased to a consistent relatively large number of publications per year.

### 3.2. Geographic Distribution and Language of TCM Scientific Production

Among the selected studies, 115 articles (10.7% of the total) did not present affiliation information, and therefore it was not possible to find out their origin. Nevertheless, the scientific production is distributed over 27 countries. China leads the ranking (816 articles; 76.1%), followed by the United States of America (32 articles; 3.0%), South Korea (22 articles; 2.1%), Japan (15 articles; 1.4%), and Germany (13 articles; 1.2%). Thirteen of these countries are in Europe, eight in Asia, two in Africa, two in North America, one in South America, and one in Oceania. Asia is the leading continent, with more than 90% of the worldwide research (see Table 1). A total of 613 articles are written in Chinese (57.2%) and 459 in English (42.8%).

### 3.3. Publication Quality

In this analysis, the impact factor was considered to be the main quality parameter. There are a large number of publications in Chinese journals (613 articles; 57.2%), ranked in the top five positions (see Table 2). Nevertheless, none of them are among the top 20 when considering the impact factor ranking (Table 3). The United States of America leads the ranking, with journals with the highest impact factors, such as The Journal of the American Medical Association (JAMA) and The American Journal of Gastroenterology, in which TCM studies were published.

### 3.4. Type of Study Design and Author’s Affiliation

In 149 (13.9%) of the 1072 selected articles, the experimental designs are not declared. Of the remainder, 4% are controlled clinical trials and 96% are randomized, controlled trials.

Due to the vast list of institutions to which the authors of the 1072 articles are affiliated, it was decided to analyze data considering specific keywords found in the affiliation section of the mentioned publications. Thus, 20.6% of the articles mention “Traditional Chinese Medicine” in the affiliation section; 31.2% mention “University” and “Hospital”; 20.9% mention “University”; 3% mention “Traditional Chinese Medicine” and “University”; 45.4% mention “Hospital”; 7% mention “Traditional Chinese Medicine” and “Hospital”; 9.2% mention “Institute”; 1.8% mention “Traditional Chinese Medicine” and “Institute”; and 0.8% mention “Engineering”.

Additionally, the publications referred to in Table 3 were analyzed regarding the author’s affiliation, and each organization of the obtained list was searched in the whole sample. Therefore, based on the number of hits, the most relevant organizations to which the authors are affiliated are: China Academy of Chinese Medical Sciences (42 hits); Shanghai University of Traditional Chinese Medicine (32 hits); Fudan University (13 hits); Sun Yat-Sen University (9 hits); Guangzhou University of Traditional Chinese Medicine (7 hits); Chengdu University of Traditional Chinese Medicine (5 hits); China Medical University Hospital of Taichung (3 hits); Beijing University of Traditional Chinese Medicine (3 hits); University of Oxford (3 hits); University of Western Sydney (3 hits).

### 3.5. Treatment Methods Mentioned in the Keywords Section

In our sample, we found a wide list of 2007 keywords. Within this list, we identified four main groups of keywords that are, in fact, treatment methods of TCM. Those groups are phytotherapy, acupuncture, moxibustion, and dietetics. For each group, several related keywords were considered as follows: phytotherapy (phytotherapy, drugs, Chinese herbal, and herbal medicine); acupuncture (acupuncture, acupuncture analgesia, acupuncture points, acupuncture therapy, ear acupuncture, and electroacupuncture); moxibustion; dietetics (diet therapy, dietary supplements, and food hypersensitivity).

The main TCM methods of treatment mentioned in the keywords section of the selected articles are phytotherapy (55%), followed by acupuncture (40%), moxibustion (5%), and dietetics (1%).

## 4. Discussion

Our results show that in the last three decades, until 2007, the number of TCM publications experienced an exponential growth. An increasing tendency was also confirmed by Fu (2010) in a bibliometric analysis of TCM research indexed in Medline between 1964 and 2008 [16]. In 2007, a peak of scientific production was registered. This highest number of publications might be related to the Olympic Games in Beijing, which assisted in the worldwide dissemination and acceptance of TCM’s clinical and therapeutic approaches [17]. From 1999 to 2012, there was an increase from 25 to 69 in the number of countries which have adopted policies with regard to traditional medicine, as well as from 65 to 119 in the number of countries with regulation of herbal medicines [20]. After the abovementioned peak, and from 2009 on, a steady-state tendency of the annual number of TCM publications can be noticed, with an average of 66 TCM articles published per year and indexed in PubMed.

Considering the publications’ geographical distribution, China accounted for 76.1% of clinical trials, followed by the USA with 3.0%, South Korea with 2.1%, Japan with 1.4%, and then Germany with 1.2%. Similar results were found by Huang et al. (2015) in a study involving 32,036 TCM articles [18], as well as by Liu (2017) in a bibliometric analysis performed on science citation index expanded (SCIE) [19]. In Huang’ study, China led the ranking with 47.69% of publications, followed by the USA with 5.23%, Japan with 4.5% and South Korea with 2.54%. Europe is the continent with the most prominent countries publishing in this area, followed by Asia. However, in terms of number of publications, Asia leads the ranking with China as the main worldwide highlight.

As shown, China is highlighted in terms of quantity (57.2%), and although the top 20 best-ranked journals, regarding the impact factor (IF), belong mostly to the USA and England, the authors of 71.8% of the TCM studies published in those journals are affiliated to Chinese organizations. In fact, from the 39 publications mentioned in Table 3, 28 are from China, three are from the USA, two are from Germany, and with one publication each, Australia, France, India, Italy, South Korea, and the United Kingdom. Nevertheless, in our sample, most of the Chinese papers were published in relatively lower IF journals, and in fact, of the whole sample, around 50% were written in Chinese, which limits the worldwide impact of the publications. Indeed, the remaining publications (42.8%) were written in English and published in relatively higher IF journals. These findings are in agreement with the results of Huang et al. (2015), who found that the first six positions were occupied by Chinese journals, but none of them had a declared IF according to the 2013 Journal Citation Reports (JCR) [18].

The period of time considered in this study covered more than three decades, during which, based on the analysis of the publication year and impact factor of the twenty best-ranked journals referred to in Table 3, an increase in quality of the published TCM articles can be reported. In fact, 53.8% of the articles published in higher impact factor journals were accepted during the last decade; 43.6% were accepted during the previous decade, and just 2.6% before that.

In our study, we observed that 96% of the publications were randomized and controlled clinical trials, which shows concern about the importance of clinical evidence in this field. In fact, the tendency to clinical-oriented research in this field was previously pointed out by Fu (2010) [16], and now confirmed by our results.

The majority of scientific publications are produced by authors affiliated to universities (20.9%), out of which 3% are TCM universities; as well as to hospitals (45.4%), out of which 7% are TCM hospitals.

With regard to the treatment methods mentioned in the keywords section of the articles, phytotherapy was the most used approach, with 55% of the references, followed by acupuncture (40%) and moxibustion (5%). Our results are in accordance with Gao et al. (2013) [17], who found that pharmacology led the research ranking in six Chinese universities, as well as with Huang et al. (2015) and Fu (2010), who showed that over the last decades, TCM research is mainly focused on pharmacology and on the therapeutic use of Chinese herbal medicine [16,18].

*Qigong*, *Tai Chi*, and *Tuina* are also methods of treatment based on the theoretical principles of TCM [21,22]; however, no citations were found in the keywords search within our sample. This fact could be due to the MeSH searching structure. Articles indexed to MeSH with the description “Traditional Chinese Medicine” do not use the keywords: *Qigong*, *Tai Chi*, or *Tuina*. In addition, the alphabetical and hierarchical structure of MeSH does not correlate the descriptors “Traditional Chinese Medicine”, “*Qigong*”, and “*Tai Chi*”. In fact, these terms are placed in different hierarchical roots. Another remark is that “*Tuina*” is not a valid descriptor for MeSH indexing. It is also important to refer to the fact that occasionally, due to the difficulty of publishing TCM-related articles in high impact factor journals, the authors opt to present their studies with a more conventional title, even if the core subject is related to Chinese medicine.

## 5. Conclusions

In summary, the increasing demand for TCM seems to be due to factors such as lower side effects and greater efficacy in some patients not responding well to conventional therapy. As a result, a considerable amount of TCM science-based literature has been produced, supporting the rational integration of these practices in Western healthcare systems and research.

Acupuncture, as one of the most popular treatment methods of TCM, is often referred to when Chinese medicine is the subject of a conversation; however, our results show that over the last decades, a higher incidence of studies on phytotherapy can be found on the PubMed database. Nevertheless, acupuncture is still widely studied by researchers all over the world.

The authors of the TCM publications selected as a sample in this study are mainly affiliated to universities and hospitals, among which some specialized TCM universities and hospitals can be found. Some authors are also affiliated to engineering departments and institutes, which denotes the transversal character of the research performed in this field, and the will to find out measurement systems and quantification strategies in order to objectify TCM variables.

Accordingly, the abovementioned integration of TCM in Western healthcare systems and research is strictly dependent on the objectivation of TCM, supported by a clear science-based definition of core concepts, diagnostic methods, and treatment procedures, as well as by scientific peer-reviewed publications in which the effectiveness of such practices must be shown. Indeed, our results show that the quality of TCM research and inherent publications has been increasing over the last decades, with a higher incidence of studies published in well-ranked journals.

In this scenario, a reference must be made to the Portuguese political-governmental and academic effort that in the last decades, pushed out the systematization, documentation, transmission, and dissemination of TCM. In fact, since 2008, the Institute of Biomedical Sciences Abel Salazar of the University of Porto (ICBAS-UP) has established the first Master’s degree in traditional Chinese medicine taught in a medical faculty of a government-funded university; the only one accredited by the Bologna process and by the Portuguese Ministry of Science, Technology and Higher Education [23], and following the proposals and recommendations of the World Health Organization.

In order to stimulate a similar worldwide opening, TCM researchers should be encouraged to carry out original and systematized scientific studies for publication in high impact factor journals. This is the way to overcome cultural-sectorial resistances rooted in our society and start a new age of healthy multidisciplinary professional cooperation focused on the wellbeing of the patient.

## Figures and Tables

**Table 1:** Ranking of TCM scientific production by countries.

Ranking	Country	Number of Publications	Percentage
1	China	816	76.12%
2	USA	32	2.99%
3	South Korea	22	2.05%
4	Japan	15	1.40%
5	Germany	13	1.21%
6	Australia	12	1.12%
7	Italy	7	0.65%
8	United Kingdom	7	0.65%
9	Singapore	6	0.56%
10	Norway	4	0.37%
11	Portugal	3	0.28%
12	Egypt	2	0.19%
13	Iran	2	0.19%
14	Austria	2	0.19%
15	Denmark	2	0.19%
16	South Africa	1	0.09%
17	Canada	1	0.09%
18	Azerbaijan	1	0.09%
19	Pakistan	1	0.09%
20	Thailand	1	0.09%
21	Brazil	1	0.09%
22	Belarus	1	0.09%
23	France	1	0.09%
24	Netherlands	1	0.09%
25	Spain	1	0.09%
26	Sweden	1	0.09%
27	Switzerland	1	0.09%
	* unknown	115	10.73%
	TOTAL	1072	100%

* Articles without affiliation information.

**Table 2:** Ranking of journals with TCM publications by country.

Ranking	Journals	Number of Publications	Percentage	Country
1	*Zhongguo Zhong Xi Yi Jie He Za Zhi*	241	22.48%	China
2	*Zhongguo Zhen Jiu*	189	17.63%	China
3	*J Tradit Chin Med*	68	6.34%	China
4	*Chin J Integr Med*	59	5.50%	China
5	*Zhong Xi Yi Jie He Xue Bao*	56	5.22%	China
6	*J Altern Complement Med*	39	3.64%	USA
7	*Am J Chin Med*	30	2.80%	Singapore
8	*Zhongguo Zhong Yao Za Zhi*	24	2.24%	China
9	*Trials*	21	1.96%	England
10	*Complement Ther Med*	15	1.40%	Scotland
11	*Zhonghua Nan Ke Xue*	14	1.31%	China
12	*Zhen Ci Yan Jiu*	13	1.21%	China
13	*BMC Complement Altern Med*	12	1.12%	England
14	*Zhongguo Gu Shang*	12	1.12%	China
15	*Zhong Xi Yi Jie He Za Zhi*	10	0.93%	China
16	*J Ethnopharmacol*	9	0.84%	Ireland
17	*World J Gastroenterol*	9	0.84%	USA
18	*PLoS One*	8	0.75%	USA
19	*Int J Neurosci*	6	0.56%	England
20	*Acupunct Med*	5	0.47%	England

**Table 3:** Ranking of journals with TCM publications, based on the impact factor.

Ranking	Journals	Impact Factor 2015 *	Country	Number of Publications	Publication Year(s)
1	*JAMA*	7.48	USA	2	1998
2	*Am J Gastroenterol*	5.86	USA	2	2006; 2011
3	*Diabetes Obes Metab*	5.70	England	1	2013
4	*Diabetes Care*	5.55	USA	1	2001
5	*Sci Rep*	5.47	England	1	2014
6	*Pain*	5.08	USA	1	2012
7	*Br J Cancer*	4.66	England	1	2012
8	*Eur J Endocrinol*	4.60	England	1	2005
9	*Fertil Steril*	4.23	USA	3	2006
10	*Aliment Pharmacol Ther*	4.09	England	1	2012
11	*Medicine (Baltimore)*	4.09	USA	1	2015
12	*Atherosclerosis*	3.92	Ireland	1	2010
13	*J Transl Med*	3.92	England	1	2015
14	*IEEE Trans Biomed Eng*	3.84	USA	1	2004
15	*Osteoarthritis Cartilage*	3.82	England	1	2015
16	*World J Gastroenterol*	3.80	USA	9	2003–2012
17	*J Arthroplasty*	3.55	USA	1	2013
18	*PLoS One*	3.54	USA	8	2007–2015
19	*BMJ*	3.47	England	1	2006
20	*Diabete Metab*	3.46	France	1	1995

* Assessed on the ResearchGate platform.
